# Supplementary material for: Expression of Oxidative Stress and Inflammation-Related Genes in Nasal Mucosa and Nasal Polyps from Patients with Chronic Rhinosinusitis
Source: Int J Mol Sci. 2022 May 15;23(10):5521. doi: 10.3390/ijms23105521 (PMC9145877; doi:10.3390/ijms23105521)
Supplement: Supplementary file 1 [file ijms-23-05521-s001.zip › ijms-1707010-supplementary.pdf]

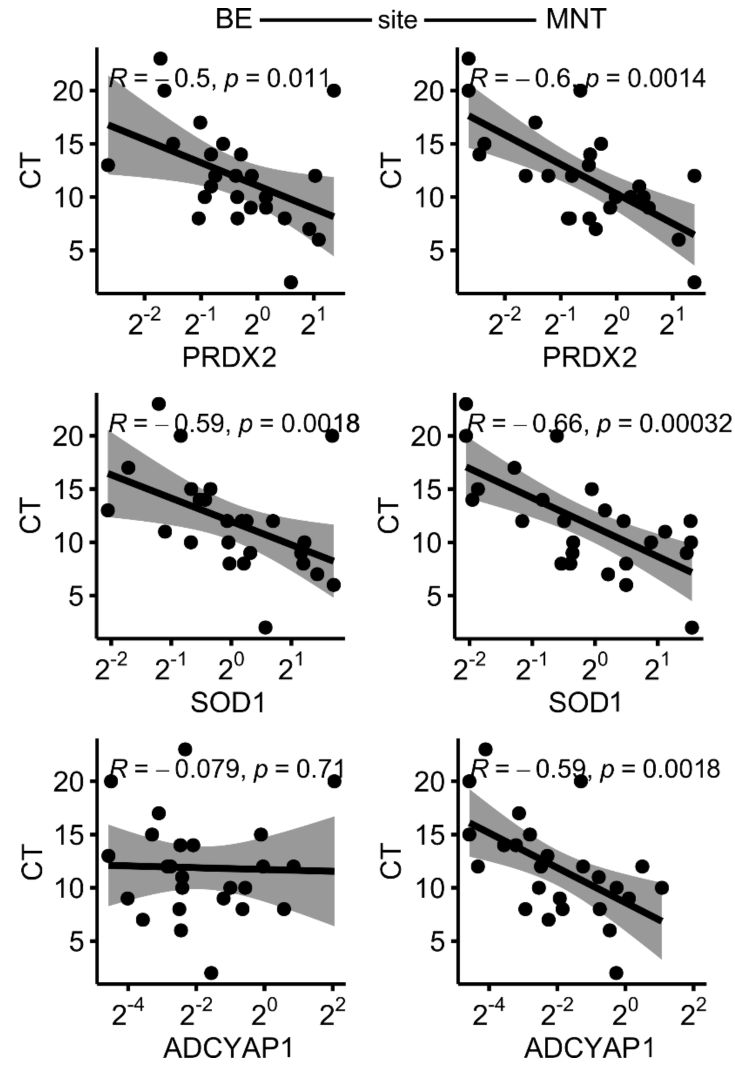

Supplementary Figure S1. Scatterplots showing co-variations between indicated gene expression (fold change, log2-scale) and Lund-Mackay CT scores. R denotes Spearman's correlation coefficient. The black line represents a linear model fit whereas the shaded region indicates 95% confidence interval. Each dot represents an individual. BE bulla ethmoidalis, MNT middle nasal turbinate.

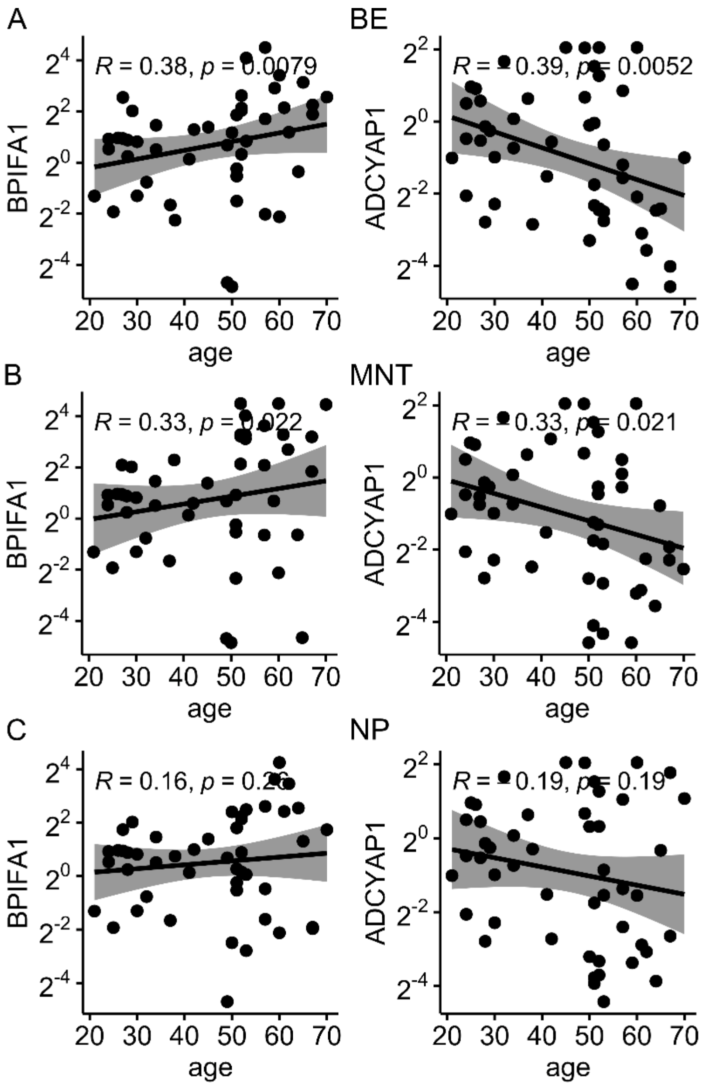

Supplementary Figure S2. Scatterplots showing co-variations between indicated gene expression (fold change, log2-scale) and age (years). R denotes Spearman's correlation coefficient. The black line represents a linear model fit whereas the shaded region indicates 95% confidence interval. Each dot represents an individual. BE bulla ethmoidalis, MNT middle nasal turbinate, NP nasal polyp.

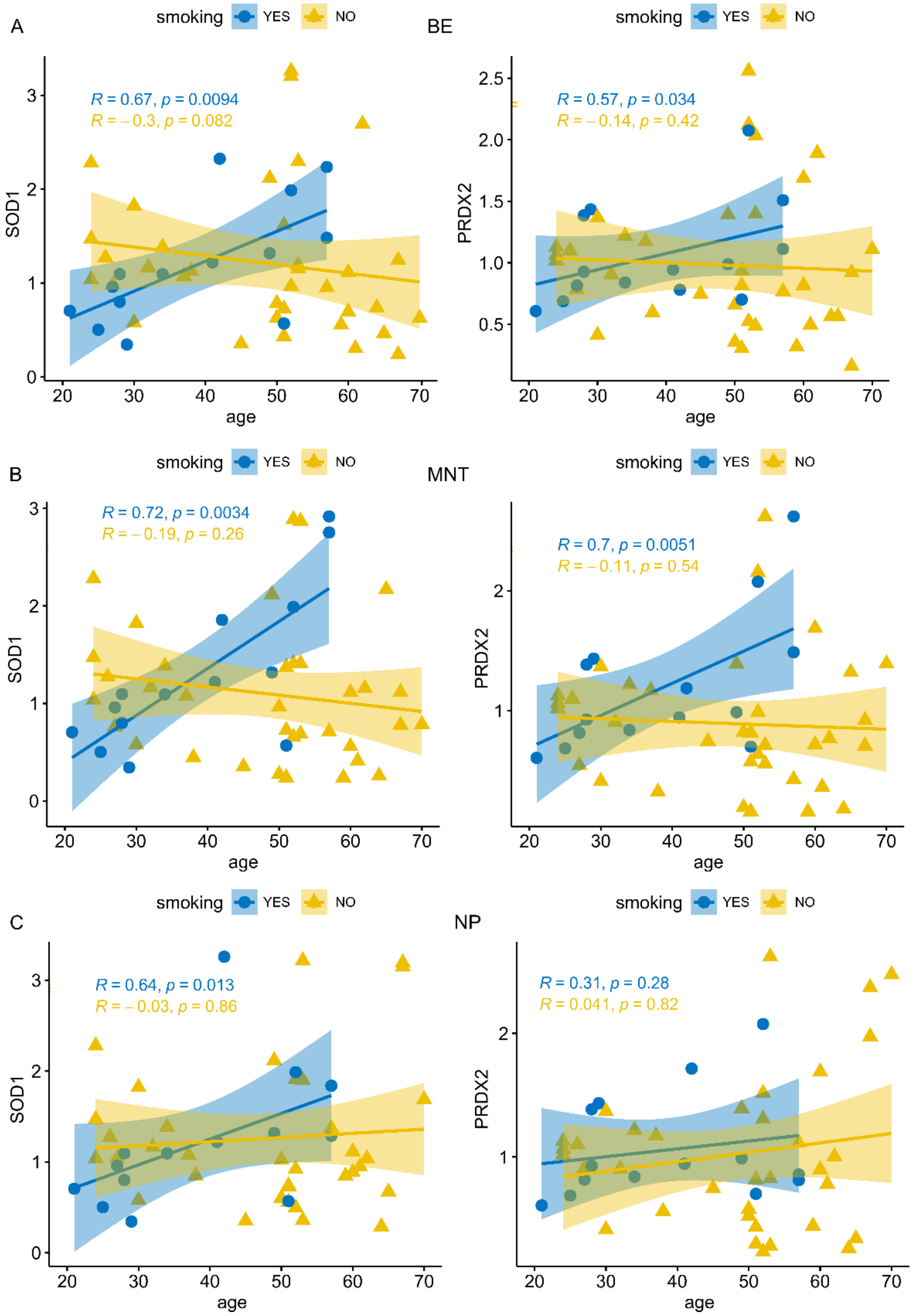

Supplementary Figure S3. Scatterplots showing co-variations between indicated gene expression (fold change) and age (years) according to smoking status
(YES/NO). R denotes Spearman's correlation coefficient. Colored line represents a linear model fit whereas the shaded region indicates 95% confidence interval. Each dot represents an individual. BE bulla ethmoidalis, MNT middle nasal turbinate, NP nasal polyp.

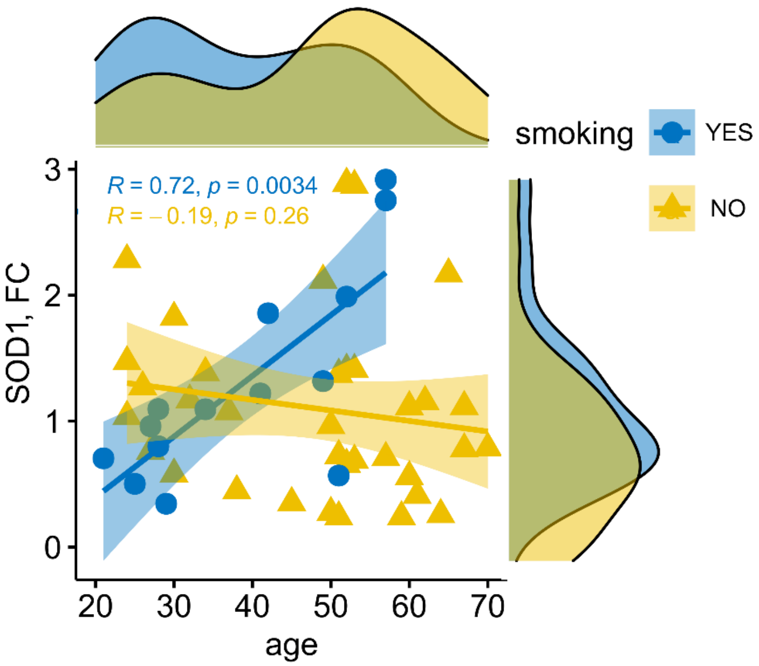

Supplementary Figure S4. Scatterplot of SOD1 [fold change (FC), middle nasal turbinate] and age (years) with their marginal density functions according to smoking status (YES/NO). R denotes Spearman's correlation coefficient. Colored line represents a linear model fit whereas the shaded region indicates 95% confidence interval. Each dot represents an individual.
